# An absence of evidence breeds contempt: A qualitative study of health system stakeholder perceptions of the quality of medicines available in Senegal

**DOI:** 10.1371/journal.pgph.0002004

**Published:** 2023-07-12

**Authors:** Mirza Lalani, Scott Kaba Matafwali, Aminata Dior Ndiaye, Jayne Webster, Sian E. Clarke, Harparkash Kaur

**Affiliations:** 1 Department of Health Services Research and Policy, London School of Hygiene and Tropical Medicine, London, United Kingdom; 2 Department of Clinical Research, London School of Hygiene and Tropical Medicine, London, United Kingdom; 3 Department of Sociology, University Cheikh Anta Diop, Dakar, Senegal; 4 Department of Disease Control, London School of Hygiene and Tropical Medicine, London, United Kingdom; 5 Institut de Recherche pour le Développement (IRD), Campus Universitaire IRD-UCAD de Hann Maristes, Dakar, Senegal; Muhlenberg College, UNITED STATES

## Abstract

Poor-quality medicines pose a significant challenge for health systems in low- to middle-income countries (LMICs),with recent deaths in multiple countries following ingestion of substandard cough syrups emphasising the need for quality-assurance of medicines in our increasingly interconnected global markets. Research also suggests that the source (country of manufacture) and type of medicine (generic/brand) are perceived to be associated with medicine quality. This study explores perceptions of medicines quality among national stakeholders of a medicines quality assurance system (MQAS) in sub-Saharan Africa. Through semi-structured interviews (n = 29) with managers from organisations responsible for the MQAS, public-sector doctors and nurses, and regulated private-sector pharmacists in three urban centres in Senegal in 2013. A thematic approach to analysis was undertaken with themes organised under three main categories, the source of drugs, the type of medicine, and medicines storage. A key emerging theme was the perception of the inferior quality of generic medicines, especially those produced in Asia and Africa, as they were lower in cost and thus believed to be less effective in alleviating symptoms than their brand versions. Medicines in Senegal’s less regulated (informal) street markets were also thought to be of poor-quality as they were not subjected to national regulatory processes or stored appropriately, resulting in exposure to direct sunlight and high temperatures. In contrast, the interviewees expressed confidence in medicines quality within the regulated sectors (public and private retail pharmacies) attributed to stringent national medicines regulation, secure medicines supply chains and adequate technical capacity to survey and analyse for medicines quality. Also, the views expressed typically described a medicine’s quality in terms of its effectiveness in alleviating the symptoms of ill health (efficacy of a medicine).These perceptions may have implications for developing national medicines policy, the procurement and supply of affordable medicines and consumers’ decision-making when purchasing medicines. Indeed, a proclivity for supplying and purchasing more expensive brand medicines may act as a barrier to accessing essential medicines.

## Introduction

The impact of poor quality medicines on public health has not as yet been investigated fully, but a World Health Organization (WHO) impact model estimated that incremental deaths in sub-Saharan Africa due to poor quality antimalarials account for approximately 2.1% to 4.9% of total malaria deaths, or approximately 3.8% to 8.9% of malaria deaths relating to patients seeking treatment [1]. Recent reports of cough syrups deaths draw widespread attention to the risks, but are rarely quantified systematically [2, 3]. Such findings illustrate that failing to assure the quality of medicines may have significant clinical and public health implications.

Target 3.8 of the WHO’s Sustainable Development Goals leading to 2030 stipulates *‘access to quality essential healthcare services and access to safe*, *effective*, *quality and affordable essential medicines and vaccines for all”* [4]. However, studies of medicine quality conducted in low-middle income countries (LMICs) indicate that there are poor-quality medicines in circulation, posing a threat to achieving this goal [5]. Poor quality medicines result from numerous contributory factors, but the mode of manufacture is a particularly prominent issue. Medicines that have been fraudulently produced to mimic an authentic (falsified) formulation or manufactured poorly (substandard) comprise the vast majority of the poor-quality medicines that have been detected [6].

In May 2017, the World Health Assembly (WHA) Member State mechanism agreed on definitions that categorise medicines quality as falsified, substandard and unlicensed/unregistered [7]. Poor quality medicines that are deliberately produced are often falsified medicines that closely mimic authentic products with intentionally fake but convincing packaging, which may be fraudulently mislabelled with respect to their identity or source [8]. Additionally a falsified medicine will not contain the stated active pharmaceutical ingredient (SAPI), and sometimes may contain another compound which may be toxic. Substandard medicines differ from falsified medicines in that they are genuine medicines that have been poorly manufactured (without intent), where the chemical content and pharmaceutical properties of a medicine do not meet the quality specifications set for them [9]. An acceptable quality medicine should be within the specifications stated in its drug monograph detailed in an international pharmacopoeia [10–12]. Definitions are often centred on the results of content analysis to measure the amount of SAPI and calculating the percentage of the SAPI stated on the label or packaging of a medicine.

In contrast, health professionals, medicine sellers and consumers describe medicines quality more in terms of clinical effects such as efficacy and safety. From the perspective of the treatment provider, medicine selection is sometimes a consideration of the benefits and risks of the treatment influenced by numerous factors including, but not restricted to, local policy and clinical guidelines, cost and clinical knowledge (e.g. of adverse effects) and experience (previous success with the treatment) [13].

Indeed, studies in LMICs [8, 9, 14] exploring these perceptions have revealed that a medicine was characterised as ‘good quality’, if it alleviated symptoms with minimal side effects (efficacy and safety), if it was a recognised brand name medicine (manufactured by a reputable company) and if it was high in cost. Furthermore, treatment providers tended to express greater confidence in the quality of medicines made in Europe compared to those stated to be manufactured in Asia and Africa [8, 10, 11]. In high-income settings, studies involving health professionals have expressed mixed perceptions about the quality of generic medicines with regard to the experience of patients, clinical effectiveness and manufacturing quality [12, 13].

At the strategic and operational levels of medicine regulatory and quality assurance systems in LMICs, there is limited information on views regarding medicine quality. The perceptions of individuals at these levels are relevant as they may shape approaches to medicine quality assurance. This may include their motivation to address pertinent issues, their specific focus on certain components of the system, their overall confidence in the medicine quality assurance system (MQAS) and subsequently, the quality of medicines controlled by the regulatory system. In this study, we explore the perceptions of the understanding of medicines quality by a range of health system stakeholders, including representatives of the MQAS authorities and treatment providers.

## Methods

### Study setting and participants

This study was conducted in Senegal from April-May 2013 in the capital city Dakar and two nearby towns, Thies and Mbour, which are representative of urban centres outside of the capital. Senegal established a Medicines Quality Assurance System (MQAS) in the early 2000s and has subsequently developed a system that undertakes periodic sampling and quality assurance of medicines primarily at the point of care i.e. post-marketing surveillance [15]. In 2009, the Ministry of Health (MoH) established a medicines quality surveillance coordinating committee. This committee included the Central Medical Stores (primary distributor of medicines to the public health sector) [16], the National Medicines Regulatory Agency (NMRA) and the Medicines Quality Control Laboratory (equipped to analyse medicines), as well as the national agencies tasked with overseeing the control and treatment of tuberculosis, HIV and malaria. Funding, logistical and technical support for the MQAS has been provided by the United States Agency for International Development (USAID) through its implementing partner, the United States Pharmacopeia (USP) [17]. Medicine quality surveillance teams in Senegal have been using the Global Pharma Health Fund MiniLab [18] to screen the quality of medicines since 2004 at sentinel site laboratories, with confirmatory analysis using methods such as the ‘gold standard’ High-Performance Liquid Chromatography (HPLC), taking place at the Medicines Quality Control Laboratory (MQCL) in Dakar.

Stakeholders of several organisations involved in medicine quality monitoring, as well as treatment providers and pharmacists, were recruited to the study and made aware of the purpose of the research. The former group comprised representatives of various national government agencies responsible for medicines procurement, distribution, regulation and quality assurance. We also interviewed persons from key external partners, including the USP. Treatment providers included doctors and nurses from government-owned health facilities (public sector). Pharmacists were recruited from both public health facilities and private sector medicine outlets.

### Study procedure and sampling

Relevant organisations were identified through discussion with research partners in Senegal as the key stakeholder authorities of the MQAS. Individuals from each of these authorities as well as former employees were purposively selected for the study to represent the views of those primarily responsible for the operation of the system. Interviews were conducted with treatment providers, including district health officers, local health clinic officers and private pharmacy owners (pharmacists) in Dakar, Thies and Mbour. A list of all public outlets was obtained from the National Medicines Regulatory Agency but was realised to be outdated and incomplete at the time of this study. Hence, outlets were purposively selected (in the selected districts, sampling from different levels of the health system, including a district hospital, large health clinics (*centre des sante*) and smaller health facilities (*poste de sante*). Given that there were around 500 private pharmacies (personal communication 2013) in Dakar alone, we opted to randomly select participants (using a random number generator) from a list of pharmacies provided by the National Pharmacy Board.

### Data collection and analysis

Semi-structured interviews (n = 29) were conducted in English or French with 13 representatives of the MQAS authorities and 16 treatment providers: doctors (n = 3), nurses (n = 2) and pharmacists (n = 11). All of the MQAS authority representatives or public sector treatment providers approached agreed to be interviewed. However, of the 23 pharmacists operating in the private sector that were initially selected for an interview, just 11 agreed to participate. Amongst those giving a reason for refusal, most cited being unavailable due to prior commitments.

Interviews lasted between 30–90 minutes and were usually held at the interviewee’s place of work, in a private office with only the participant and researchers present or were conducted over Skype. Written informed consent was obtained from each participant prior to the interview. Interviews were conducted by ML, a pharmacist in the UK with experience in undertaking qualitative interviews in low-income settings, and ADN, a medical anthropology student with experience working on research projects in Senegal. No follow-up interviews were conducted.

Interview guides were not formally piloted prior to use but were reviewed by local collaborators who had a sound understanding of medicines quality and the MQAS in Senegal. Interview guides were designed to be iterative, allowing exploration of emergent issues from the interviews. Interview guides covered several topics, including perceptions of the quality of medicines in Senegal; medicines quality surveillance (including its functions, operational strengths and weaknesses and overall effectiveness in assuring medicines quality); and the perceived national situation in terms of poor-quality medicines. That said, an inductive approach was taken throughout the study, field notes were discussed and reviewed by both ML and ADN at the completion of each interview, and emergent themes from one interview were then incorporated for further exploration in subsequent interviews [19]. Interviews continued to be conducted after thematic saturation was reached to ensure that perspectives from the diverse range of stakeholders were captured and not overlooked.

All interviews were audio recorded and then transcribed verbatim in the language they were recorded in. Interviews conducted in French were transcribed and then translated into English. Interview data was managed using NVivo version 10. An individual external to the research team was employed to undertake translation. The quality of translation of 3–4 transcripts was verified by ADN. Thematic analysis, an approach to code the data and identify patterns and themes, was undertaken [20]. A sample of transcripts were coded independently by ML, and ADN, and resulting emerging themes were discussed to create a thematic framework. This framework was also discussed with the other co-authors to achieve further agreement [20].

### Ethics considerations

Approval was obtained from the London School of Hygiene and Tropical Medicine Research Ethics Committee (reference no. 6330) and the National Committee of Health Research (Senegal, reference no. 0045). Written informed consent to participate was obtained from participants.

## Results

Our analysis of the interview data revealed three overarching themes relating to the perceptions of medicine quality: 1) the source of medicines, 2) generic and brand medicines, and 3) the impact of medicines storage on quality.

### Source of medicines

Perceptions relating to the source of medicines and their quality included: the stated country of manufacture or origin, and the informal (unregulated) and regulated (public and private) sectors.

### Stated country of manufacture/origin

All interviewees mentioned a preference for medicines stated as manufactured by European and North American companies as these were assumed to be of reliable quality. This perception was predicated on three main aspects: 1) these medicines were produced by reputable manufacturers, 2) the medicines were thought to undergo strict regulatory controls in the country of manufacture before export, and 3) they must be of a high standard since these medicines were the same as those consumed by European and American citizens. In contrast, treatment providers expressed more negative views of medicines manufactured in India, China and Nigeria, citing that these posed a risk to medicines quality.

*‘I was in the rural area Vélingara*, *where you can see many products entering Senegal…There were a lot of fake products coming from Nigeria……*. *I do not know if all of them are not of good quality because*, *as they say*, *drugs come from Asian countries like India and China*, *but why not from France*, *Belgium*, *which are countries that have the means to control these products*.*’* Treatment provider 6 (private sector)

### Unregulated sector

Senegal has a large unregulated (informal) sector in the form of vast open markets, such as Keur Serigne Bi in Dakar and similar locales in other principal cities, such as Touba [21]. There was a general consensus that medicines available in this sector were more likely to be of poor quality for two main reasons. Firstly, the conditions under which medicines were stored here were deemed inadequate as they were often available as loose tablets in transparent plastic bags and exposed to direct sunlight and high temperatures for prolonged periods. Secondly, the origin and status of the medicines available in this sector were questionable as medicines may have been brought into the country illegally and thus not subject to national regulatory measures.

*‘Drugs sold in this market are of poor quality*. *When you go to Keur Serigne Bi you see drugs exposed to the sun*, *lying on the ground which may damage them…They are not registered*, *we know nothing about the origin*, *who manufactured them and when they made them*.*’* MQAS authority representative, senior manager 1

Many stakeholders expressed concerns that the MQCL only collected samples from the regulated sectors as part of post-marketing surveillance activities and not from the unregulated informal sector. Two main reasons were provided for not sampling from the informal sector. Firstly, MQAS authority representatives were concerned that if good quality medicines were found in the informal sector, this may inadvertently legitimise their trade, creating a false perception among the public that medicine quality is acceptable in this sector. Secondly, the socio-cultural context within which the informal sector operated was a possible barrier to sampling. Senegal is a predominantly Islamic country in which religious brotherhoods hold a key role in society and politics. These brotherhoods were thought to be the main proponents of the informal sector, operating a large proportion of street markets and employing people from the lowest socio-economic groups. A few interviewees suggested that apprehending operators and disrupting informal sector trade may have political ramifications for government officials who relied on support from such brotherhoods. One interviewee mentioned that targeting the informal sector may be regarded as discriminatory as the sector catered to and employed people from the lowest socio-economic group in society.

‘*The politics involved in the informal sector made it very tricky*, *for the ministry to regulate the informal sector*. *It was clear that this sector had the ability to scare off ministry people through their connections*. *Also*, *if you think about it*, *if you are riding hard on the informal market you were seen as kind of an elitist*.*’* MQAS authority representative, senior manager 3

### Regulated sectors (public and private)

The perception of medicines quality available in the regulated sectors was in stark contrast to the informal sector. All stakeholders were confident in medicines quality in the public and regulated private sector which was attributed to perceived stringent national medicines regulation and secure medicines supply chains. In particular, the source of medicines (manufacturers and wholesalers) supplying these sectors were deemed trustworthy.

‘*You know the tenders were done*, *there was better traceability*, *you know who was making the drug and providing it and so the public sector I felt really comfortable*, *the private sector–quite comfortable*, *the illicit sector–not very comfortable*.*’* MQAS authority representative, senior manager 8

Some interviewees also mentioned the role of the national MQCL in assuring medicines quality as a key component of the national medicine quality assurance strategy, especially the systematic approach to medicines sampling in post-marketing surveillance whereby certain medicines classes (e.g. antimalarials) were collected on a regular basis. The standardised sampling approach was coupled with robust analytical techniques employed to detect medicines which together produced valid and reliable results on the quality of medicines available nationally.

### Generic and brand medicines

Perceptions about medicine quality were also expressed in relation to the type of medicine, i.e. generic or brand, and its cost. Generic medicines were widely available in both public and regulated private sectors and were generally cheaper than their brand versions, and the majority were manufactured in India and China. Most interviewees doubted the quality of generics. Treatment providers suggested that patients were also less trusting of generics, perceiving them as less efficacious than the brand version. The lack of familiarity with the ‘name’ of a generic and the stated manufacturer raised doubts with all interviewees and reportedly with patients. Treatment providers also mentioned that patients associated quality with cost, perceiving more expensive drugs to be more effective at treating their symptoms. Several interviewees provided specific examples of supposedly ineffective generic medicines which compromised patient safety. The most frequently cited was that of diazepam (a benzodiazepine with anxiolytic and sedative properties).

‘*If I take diazepam for example which is a drug that is prescribed against convulsions in children*, *it has since been said that it does not work in generic form properly*. *When you put it on a convulsing child*, *he would still convulse*: *whereas if you give him Valium*, *convulsion would stop*.*’* MQAS authority representative, middle manager 1

### Impact of medicines storage on quality

All interviewees mentioned that medicine quality was affected by conditions of storage (especially at the outlet level). This is a pervasive issue in the tropics, given high temperatures and humidity, especially in rural areas where power supplies may be intermittent [22]. Medicines quality was assumed to be acceptable when they arrived at a facility, but that improper storage conditions would contribute to their degradation. Despite this being a major concern, interviewees believed solutions such as installing cooling systems to regulate temperature and humidity lacked feasibility due to their prohibitive costs, especially for smaller facilities.

‘*We realised that the quality of the drugs stored at the central medical stores was better than the quality of drugs stored in health facilities*. *This means that the environment*, *the temperature etc*. *at the facility has an impact on the quality*.’ *But individual facilities cannot pay for equipment to make the temperature acceptable*.*’* MQSS authority representative, middle manager 4

## Discussion

The study revealed a wide variety of perceptions relating to the quality of medicines available in Senegal, but all stakeholders agreed that medicine quality is an important consideration. The stakeholders interviewed typically framed medicine quality in terms of its efficacy (the ability of the medicine to alleviate physical symptoms), in contrast to the formal WHO definitions which are based on chemical content, packaging and label claims. The findings shed light on several contrasting assumptions: on how generic medicines are believed to be inferior in quality to their brand versions, that medicines from the ‘east’ (China/India) are perceived less favourably than those from Europe and North America and that medicines available in the informal sector are more likely to be of poor quality. Furthermore, the current approaches to storing medicines in the tropics were thought to profoundly affect medicines quality.

A strength of this study was that the data for analysis was drawn from a broad sample of stakeholders within the MQAS, including representatives holding strategic and operational roles within the authorities responsible for operating the system. A limitation of the study was that perceptions of the public with regard to medicines quality were not captured, although we did interview a randomly selected sample of pharmacists, the main suppliers of medicines to the public in Senegal at the point of care [21]. The views of those who sell or supply medicines at the point of care and the public are important as they provide insights into how demand for medicines maybe be influenced by perceptions of quality. Furthermore, this study does not include perceptions of informal medicine sellers. Nevertheless, we interviewed several treatment providers and pharmacists from the regulated sectors. Regulated private pharmacies are abundant in both urban and rural Senegal, increasing access to essential medicines for the local population [21]. We recognise that the study findings are from a decade ago but have been assured through recent literature searches that they remain pertinent. Since this time, donors and governments have continued to invest in surveillance systems, and there may have been improvements in medicine quality assurance systems, increased access to higher-quality generic medicines, and more stringent regulation in LMICs, including Senegal. However, without more recent studies or data, it is difficult to accurately assess the current situation. Hence, this study can serve as a baseline for further comprehensive research to investigate the perceptions of medicine quality in LMICs, for which there remains limited published data, particularly from the perspective of national actors, which has implications for policymaking and the improvement of healthcare systems in these countries.

Previous studies examining perceptions of medicines quality in LMICs have primarily sought views from treatment providers and consumers, seldom exploring the beliefs of national policymakers and regulators, which are crucial to shaping the national medicines quality agenda. In this study, we sought to capture the views of senior representatives of the MQAS. Hence, the findings in this study are transferable to other health systems in sub-Saharan Africa as Senegal shares similar features, especially in those countries where an MQAS exists. Firstly, Senegal is one of several sub-Saharan African countries supported by the USP Promoting the Quality of Medicines Programme [17]. Secondly, Senegal imports most of its medicines (including generics) from India and China. Finally, Senegal has a reportedly burgeoning informal sector for medicines [23]. We have also presented perceptions of the quality of medicines in the informal sector that have rarely been discussed in the research literature. The informal health sector in sub-Saharan Africa provides a convenient alternative for consumers, especially in locations where regulated health facilities (public or private) are difficult to access [24]. Perceptions of the public regarding medicine quality have been discussed elsewhere [9, 14]. Understanding public perception is essential as it can be used to inform public awareness campaigns educating the population on the scarcity of evidence to prove factors such as origin/source, cost, and type (generic/brand) affect medicine quality and that the vast majority of generics are safe and effective as they are approved by the WHO prequalification programme or similar initiatives [25].

Several factors may be considered by treatment providers and consumers when supplying or purchasing medicine [26]. We found that the stated country of manufacture (source) of medicine was viewed as a proxy for quality, with suspicion of medicines made in India, China and Nigeria, which mirrors similar findings from other studies [10, 14, 27, 28]. These perceptions are corroborated by several reports of the discovery of poor-quality medicines that were stated to be manufactured in India, China and Nigeria [29–31]. Conversely, medicines stated to be from Europe, or North America were typically thought to be of good quality, echoing similar findings among consumers in urban Tanzania [32]. The view that generic medicines are inferior in quality to their brand versions has also been reported elsewhere [33, 34]. It may be difficult to disentangle perceptions of the quality of generics from concerns about the place of origin, manufacturing standards and cost.

This study also found that the informal sector was perceived to be a significant threat to medicine quality in Senegal primarily due to improper medicine storage practices and a lack of authentication of the source or origin of the medicines available. Data on medicine quality in the informal sector in Senegal is limited to just a single study conducted by USP in 2010, which found that 68% (13/19) of samples of antimalarials in the informal sector failed quality control testing in comparison to 32% (14/43) in the regulated sector [35]. The MQAS ceased sampling from the informal sector in 2010. Therefore, there were no reliable data on medicine quality from this sector at the time of our study in 2013. Our findings identify two barriers to undertaking sampling from the informal sector: fear of endorsing trade in this sector and concerns relating to the cultural and political influence of those involved in the operation of the sector. The latter issue may be unique to Senegal, yet it illustrates how moral and political considerations can play a key role in shaping surveillance systems, the research undertaken, and the resulting data available.

In contrast, with respect to both the public and regulated private sectors, interviewees were assured by the existence of national medicines regulations and medicines supply chains. A degree of this confidence was attributed to the WHO prequalification programme, which assures the ‘quality, safety and efficacy’ of medicines prior to their distribution to recipient countries [36]. Results from nationally conducted medicines surveys provide some rationale for assuredness in the MQAS, with data from a survey in 2012 showing that 93.8% of 481 antimalarial samples collected from the public and regulated private sectors passed the MiniLab screening test for medicines quality [37]. However, a notable concern for all stakeholders interviewed in this study was the effect of storage conditions on medicine quality. Medicine degradation and loss of activity may be accelerated by storage conditions such as exposure to sunlight, high temperatures (higher than 30°C) and high humidity [38]. Yet, inadequate storage was perceived as an everyday reality of the climatic situation in the tropics, and therefore there was an ambivalence about what steps could be taken to address the issue.

The study findings also provide insights into how the quality of a medicine is defined and described by those responsible for designing, implementing and enacting medicines quality policy at a national level, which differs somewhat to the formal definitions and terms used in the policy and academic literature. Lalani *et al* [39] identified three different paradigms for describing medicines quality: legal, technical and clinical. The legal paradigm focuses on any judicial process against a producer based on providing evidence that there was an intent to produce a poor-quality medicine [14]. The technical paradigm describes the chemical content and pharmaceutical properties of a medicine. The clinical paradigm frames medicine quality in terms of its effectiveness in alleviating symptoms (efficacy) with minimal adverse effects (safety), i.e. the clinical consequences of poor quality medicines on the health of a population. Respondents in this study usually frame medicine quality in terms of the clinical paradigm, which is in stark contrast to the WHA definitions, which are centred upon the legal and technical paradigms. Moreover, the majority of medicines quality studies categorise the quality of medicines detected in surveys as being quality assured (technical), falsified (legal), substandard (technical) or degraded (technical) [39]. To date, there has been little consideration of the clinical paradigm. However, it is recognised that the paradigms overlap and interact as the clinical effect of a medicine (its efficacy or safety) is dependent upon its specification, such as the amount of active pharmaceutical ingredient (technical) and the standards to which it has been manufactured (legal/technical).

## Conclusions

These study findings have important implications for national and international policymakers, national regulators, pharmaceutical companies and treatment providers in LMICs, and future medicines quality-related research. The concerns expressed by stakeholders that medicines are likely to be of poor quality if they are 1) made in Africa or Asia, 2) are generic or 3) available in the informal sector are largely unsubstantiated. Such views may undermine international efforts to increase access to affordable medicines for the world’s poor. This is contrary to the premise of the WHO essential medicines list that was established to promote the use of medicines that were listed according to their critical need in treatment [40]. These perceptions are also significant in light of the growing market share of generics in sub-Saharan Africa [41]. Hence, future research requires more medicine quality studies which use epidemiologically sound sampling methods to investigate the quality of generics in use at the point of care and quantify the scale of the problem, to provide the much-needed evidence to either substantiate or allay these widely-held concerns.

Medicine quality studies are generally conducted and reported in the technical paradigm, yet the perceptions of medicine quality do not fit this model but align better with the clinical paradigm, which characterises quality in terms of its impact on population health. This is a vital consideration for future research, which should ensure that findings and subsequent recommendations are relevant to treatment providers and the public, who are more concerned with the effectiveness and safety of the medicine they supply or consume rather than if a medicine satisfies a technical or legal standard. This study has also illustrated the complexity of the perceptions of medicine quality and presents some contradictory views. For example, generics were thought to be of inferior quality to their branded versions, yet treatment providers had confidence in the quality of medicines they sold and supplied to the public: many of which were generic products. The heterogeneity of such views challenges policymakers, pharmaceutical companies and regulators in informing treatment providers and consumers about the realities of medicine quality. Public information campaigns by national regulators and pharmaceutical companies, supported by research-based evidence of generic medicine quality, may change these perceptions.
